# Young women’s experience of personal recovery following acute myocardial infarction: A qualitative study

**DOI:** 10.1371/journal.pone.0298798

**Published:** 2025-09-09

**Authors:** Cenjing Zhu, Andrew Arakaki, Alisia Pan, Karina Danvers, Andrea Barbardo, Janan Wyatt, Catherine X. Wright, Donald S. Wright, Louise Pilote, Valeria Raparelli, Joan K. Monin, Gabriele Oettingen, Rachel P. Dreyer, Anthony J. Pavlo

**Affiliations:** 1 Department of Chronic Disease Epidemiology, Yale School of Public Health, New Haven, Connecticut, United States of America; 2 Department of Chemistry, Yale University, New Haven, Connecticut, United States of America; 3 Yale Program for Recovery and Community Health (PRCH), New Haven, Connecticut, United States of America; 4 Department of Psychiatry, Yale School of Medicine, New Haven, Connecticut, United States of America; 5 Department of Cardiovascular Medicine, Yale School of Medicine, New Haven, Connecticut, United States of America; 6 Department of Emergency Medicine, Yale School of Medicine, New Haven, Connecticut, United States of America; 7 Centre for Outcomes Research and Evaluation, Research Institute, McGill University Health Centre, Montreal, Quebec, Canada; 8 Department of Medicine and Research Institute of the McGill University Health Centre, Montreal, Quebec, Canada; 9 Department of Translational and Precision Medicine, Sapienza University of Rome, Rome, Italy; 10 Department of Social and Behavioral Science, Yale School of Public Health, New Haven, Connecticut, United States of America; 11 Department of Psychology, New York University, New York, New York, United States of America; 12 Department of Biostatistics, Yale School of Public Health, New Haven, Connecticut, United States of America; Tabba Heart Institute, PAKISTAN

## Abstract

**Background:**

Rates of acute myocardial infarction (AMI) morbidity and mortality have increased in young women aged ≤55 years but little is known about their experience recovering from and living with AMI. A personal recovery (experience of an identity shift manifested in both losses and gains) has been reported among general AMI survivors. Our objective was to gain insights into young women’s perspectives on long-term post-AMI recovery, under the patient-centered personal recovery framework.

**Methods and results:**

The study used a participatory, phenomenological approach. In-depth interviews were conducted with 18 young women (18–55 years at the time of their AMI) who were readmitted within the first year post-AMI; these were audio-recorded, transcribed verbatim, and analyzed in collaboration with two women with lived experience of AMI. Study findings revealed that young women’s experience did not deviate largely from the personal recovery framework from the general AMI survivors (i.e., an identity shift manifested with both losses and gains). However, certain aspects in the psychosocial domain were highlighted and further articulated to address young women’s experience. Specifically, within the three categories of the personal recovery framework, two of which (i.e., loss, and gain) can be further classified, eight themes were identified around the topics of loss of safety and security, self-worth, social roles, (both the loss and gain of) hope and optimism, and gain of connection, strategies to manage emotions, and meaning and purpose.

**Conclusion:**

Findings validated the utility of personal recovery framework in capturing the experience of young women with AMI, and highlighted themes that address young women’s distinguished ways of meaning-making, which anchors around social roles and personal identity. Identifying opportunities to improve awareness of self-care and facilitating social support for young women after AMI represents important targets for future intervention.

## Introduction

Despite an overall reduction in cardiovascular disease prevalence and acute myocardial infarction (AMI) deaths in the general population, rates of AMI in young adults (≤55 years), especially women, have increased over the past two decades [1–3]. Compared with similar-aged men, young women are less likely to achieve targets of physical activity recommendations [4], smoking cessation [5–7], risk factor control [8], and adherence to cardio-protective medications [9,10]. This may contribute to women experiencing worse post-AMI outcomes including poorer health status [11], more depression/stress [12,13], lower likelihood of returning to work [14] and higher rates of readmission through 1-year [15]. However, prior qualitative literature has only focused on young women’s experience with AMI symptoms and treatment during the acute phase [16–22]. The causal factors that may contribute to the higher long-term risk, including readmission, among young women with AMI remains poorly understood. Attending to these issues through a patient-centered approach to care may improve outcomes for young women.

A conceptual framework for personal recovery has been recently developed to better characterize patients’ experience of AMI recovery and distinguishes between personal and clinical recovery [23]. Personal recovery refers to a deeply personal and unique process of changing one’s attitudes, values, feelings, goals, skills and/or roles. Distinct from the traditional clinical recovery models focusing on standard secondary prevention in cardiology, this new model empowers individuals to identify a way of living satisfying, hopeful, and meaningful lives even with the limitations caused by illness [24]. The framework suggests that people experience a major identity shift as a consequence of the AMI, which manifests in both losses and gains. Both themes pose new challenges to everyday life, making their experience of recovery following AMI an active daily process that requires people to take responsibility for their own self-care. While this framework speaks to the general experience of AMI recovery, it has not been validated in younger women with AMI. Thus, it remains unknown whether the personal recovery framework captures the experience of young women with AMI and how their specific preferences and needs may explain gender differences in outcomes. This paucity in knowledge makes it difficult to develop cardiac care and appropriate interventions for younger women with AMI, a population that exhibits particularly high risk of adverse outcomes including hospital readmission [25].

Thus, the aim of this study was to use a participatory, phenomenological approach to understand young women’s personal recovery following an AMI. Findings from this study could inform future gender-tailored interventions to mitigate disparities and improve long-term outcomes for young women with AMI.

## Methods

### Study design

This study used a participatory, phenomenological approach to both elicit and understand personal recovery in younger women who have experienced an AMI [26–29]. A phenomenological approach to interviewing and analysis was most appropriate to the research question as we were interested in both the ways people derived meaning from their experience of AMI and the meanings themselves [28]. In-depth interviews with patients were conducted to collect rich, detailed narratives for generating possible understandings of recovery from the lived experience of patients post AMI. Two women who with experience of AMI medical care and cardiac rehabilitation (CR) were part of the analysis team (K.D., A.B.). All team members were trained in phenomenological qualitative methods based on the participatory work of the authors (A.J.P., R.P.D.) [23,30].

### Participants and data collection

Between February 5th and August 4th, 2021, we screened 265 young women who were between 18–55 years of age at the time of their index AMI from 3 urban and suburban sites across Yale-New Haven Health (The Health Heart and Vascular Cardiac Rehabilitation Center, the Yale-New Haven Health TakeHeart Cardiovascular Health Center, and the Yale-New Haven Health Chest Pain Center). To be eligible, patients must have had an AMI during the index hospitalization based on the Fourth Universal Definition of Myocardial Infarction [31] and have been readmitted to hospital within 1 year. Each patient chart was adjudicated separately by the two physician clinical researchers (C.X.W. and D.S.W.) for inclusion; in cases of disagreement, the specific patient chart was re-reviewed jointly for a final decision. Among 35 eligible participants, 30 were contacted and 18 were interviewed in the study before achieving saturation – further data collection did not produce value-added insights. Detailed inclusion criteria and flowchart can be found in S1 Text. Institutional review board approval was obtained at Yale University. Patients provided informed consent verbally, as the research was deemed no greater than minimal harm to patients. A trained research assistant reviewed the content in informed consent with participants. Participants were provided a copy of the informed consent document before meeting the research assistant. There was no preexisting relationship between interviewers and participants in this study.

A semi-structured interviewer guide (S2 Text) was developed with input from people with lived experience of AMI (K.D., A.B.) and researchers with experience in recovery-oriented psychiatric research (A.J.P.). Participants were interviewed via zoom by researchers trained in qualitative interview methods (J.W., R.P.D., A.J.P.). Interview length averaged around 38 minutes and varied from 18 to 55 minutes. Baseline information on demographics and socioeconomic status was collected through an online survey and patient report.

### Data analysis

The interviews were audio recorded and transcribed verbatim. Participants were not invited to review their transcripts or the findings (i.e., member checking). Our participatory approach was used to ensure that the perspectives of patients were central and that the analyses did not get subsumed by traditional, clinical understandings [27].

Qualitative data were analyzed in the following steps and involved a continuous dialectical movement between the whole and the parts of the text (i.e., between understanding and explanation). First, transcripts were read repeatedly in an open-minded way using techniques of naïve reading, and then were condensed into narrative summaries written solely in the participants’ own words in order to highlight the meaning participants made of events and experiences [29]. Second, each narrative was analyzed to identify patterns across participants. We condensed and abstracted the narrative elements, focusing on their similarities and differences to form themes and sub-themes. This process was facilitated by a consensual model implemented by 5 members of the research team (C.Z., A.A., A.P., A.J.P., R.P.D.). Last, summative themes emerged through forming a common narrative from all participant interviews. This common narrative was verified against each original transcript to ensure fidelity to participants experiences. Data were coded and managed in Dedoose (version 8.3). For quantitative data, baseline characteristics were examined for the overall population with categorical variables presented as numbers (%) and continuous variables presented as mean (SD) or median (min-max). All analyses were performed in SAS version 9.2 (SAS Institute, Cary, NC).

## Results

### Study characteristics

The 18 young women who participated in our study had a mean age of 52.0 ± 4.0 years at the time of interview. Intervals between index AMI and the interview ranged from 1 to 3 years (mean 2.0 ± 0.9 years). There were 56% non-Hispanic White (n = 10), 33% non-Hispanic Black (n = 6) and 11% Hispanic (n = 2) participants. Of the total population, 10 (56%) participants were referred to cardiac rehabilitation (CR) and 8 (44%) actually participated in CR. Demographic characteristics and clinical factors of the population are detailed in Table 1.

**Table 1:** Baseline characteristics of study population.

Participant characteristics	Total (n = 18)
Age, year	
median [min-max]	44 [39-58]
Race	
White	10 (55.6%)
Black	7 (38.9%)
Other	1 (5.6%)
Ethnicity	
Hispanic	2 (11.1%)
Education	
High school or less	3 (16.7%)
Some college	7 (38.9%)
Graduated college/graduate school	6 (33.3%)
Missing	2 (11.1%)
Marital status	
Married	7 (38.9%)
Divorced/Separated	5 (27.8%)
Single/Never married	6 (33.3%)
Employment status	
Working full time	11 (61.1%)
Working part-time/Self-employed	2 (11.1%)
Unemployed/Disabled	5 (27.8%)
AMI diagnosis	
STEMI	11 (61.1%)
NSTEMI	7 (38.9%)
Cardiac rehabilitation referral	
Yes	10 (55.6%)
No	6 (33.3%)
Missing	2 (11.1%)
Cardiac rehabilitation participation	
Yes	8 (44.4%)
No	8 (44.4%)
Missing	2 (11.1%)
Insurance	
Commercial	11 (61.1%)
Medicare/Medicaid	7 (38.9%)
Tobacco use	
Current smoker	8 (44.4%)
Former smoker	5 (27.8%)
Never smoker	5 (27.8%)
Alcohol use	
Yes	7 (38.9%)
No	10 (55.6%)
Missing	1 (5.6%)
Substance use	
Yes	4 (16.7%)
No	14 (77.8%)
Comorbidities	
Hypertension	4 (16.7%)
Type 2 Diabetes	2 (11.1%)
Hyperlipidemia	1 (5.6%)
Obesity	10 (55.6%)
Depression	3 (16.7%)
Family history of heart disease	8 (44.4%)

### Qualitative findings

**Fig 1:**
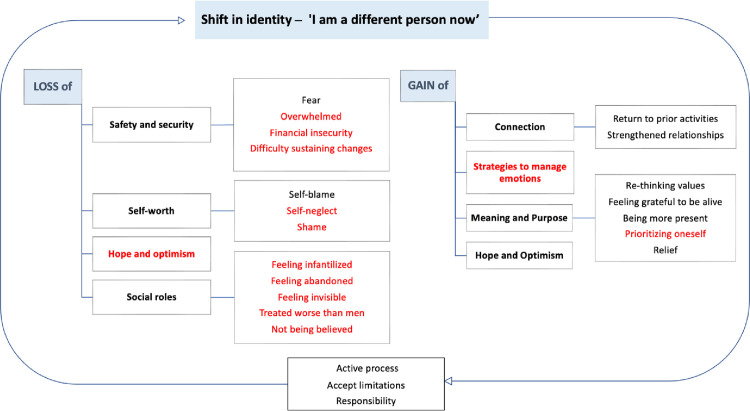
Conceptual Framework of young women’s experience recovering from AMI.

Findings from young women’s experiences recovering from AMI resulted in 8 main themes which overlapped with three main categories of the personal recovery framework (i.e., shift in identity, loss and gain) (Fig 1) [23]. Thus, the presentation of findings is centered around these three major categories, with original direct quotations presented to illustrate the unique findings and themes to illuminate young women’s experiences. Description of the same themes as those in the general framework were not elaborated upon and can be found in prior work [23]. Young women’s quotations of these common themes were included in S3 Text.

### SHIFT IN IDENTITY - ‘I’m a different person now’

Young women’s experience following an AMI is embodied in the overarching theme of feeling like a ‘different person’ during their recovery period. Two women described conflictual feelings of this shift in identity:

*“Right now, I just can’t seem to get a hang of things…You know, it’s like I can’t control my feelings and emotions. I just feel like a totally different person.” (16)*

*“I need to have fun and be present and enjoy. So, I guess you could say I found me. I found a different me.” (121)*

Young women frequently described how they were forced to take an active role in driving and managing their recovery. They found that life after an AMI entailed an unremitting endeavor to achieve normalization and they described the period after AMI as an ongoing ‘**active process**’:

*“I’ve learned that if I want to plan out my week I realized that that’s too broad of a range. I have to take it day by day [and] how I’m feeling at the start of the day will dictate what it is that I can accomplish for that day. I do my very best to set a schedule, making sure that I’m eating a light breakfast, staying hydrated during the day, making sure that I’m taking my medications.” (119)*

There were certain tasks or responsibilities that young women were no longer able to do safely or comfortably after an AMI. As a result, they had to **accept their limitations** and proactively adjust to the ‘new normal.’

*“I get tired a little bit easier, I call it feeling my bones. I’ll push myself, but I also know when my heart is telling me it’s enough. I usually wear a smartwatch and if I’m jogging and my heartbeat goes over 145, then I know I need to back it off a little bit. [In terms of daily tasks], I’m doing more now than I used to. I’m more careful with it.” (157)*

Young women also described a changed or heightened sense of **responsibility** to improve their health and maintain their social roles as a caregiver or breadwinner in their families after AMI. Specifically, women expressed a desire to improve their health in order to fulfill familial or professional duties.

*“Everything else can be at hold but when it comes to the children and the food and a roof over your head that’s what you think about, you know, just to make sure that you keep that. I’ve been lucky enough to keep a job during this epidemic.” (170)*

## Loss

Four themes were identified under the category of loss: young women described that the AMI had threatened and had negative effects on their sense of safety and security, self-worth, degree of hope and optimism, and social roles.

### Loss of safety and security

#### Feeling overwhelmed.

Compounded by constant ambiguity, fear and anxiety, young women often felt **overwhelmed and out of control**, seeking medication and psychological therapies to help manage their emotions. One women described how she dealt with her depression after the AMI and how her emotions in turn affected her recovery:

*“I need to be able to function like a normal human being without feeling so angry all the time. I want to scream or yell or cry. [I was] just not happy and not in a good space. It was hard for me to come to terms with the fact that this was something else that I was going to need to be treated for. It’s just really overwhelming with all that I have going on, and trying to maintain my heart health and not get stressed and overwhelmed with it.” (105)*

#### Financial insecurity.

Women often also found themselves struggling with finances, being financially responsible for others, and feeling **insecure with their financial situation**. One woman described how she was financially constrained and had to go back to work despite doctors’ suggestion for attending CR.

*“Yeah, I have to go back to work. There was no question or even if the doctor told me that I couldn’t go back to work there was no -- the bills can’t get paid without me working. It is difficult for somebody like me that doesn’t have a support system to be able to come out from the hospital and then, you know, have this activities that you’re supposed to have, but then cannot because of your financial situation or whatever situation you might have. Everything else can be at hold but when it comes to the children and the food and a roof over your head that’s what you think about, you know, just to make sure that you keep that.” (170)*

#### Difficulty sustaining changes without support.

Young women also reflected on other barriers, such as the lack of support in their social network, which made it **difficult to sustain lifestyle changes** and thus added to their sense of insecurity:

*“I did try and quit smoking for about three months, you know, after I came out of the hospital, but then I started hanging out with the people that still smoke and, that came back around and, it’s hard. It’s really, really hard and fortunately, I don’t have anybody that smokes in the house, but then my coworkers and friends are smoking. You work with them or you, you know, so yeah, it doesn’t help” (170)*

### Loss of self-worth

#### Self-neglect.

Young women also **ignored themselves** post AMI, neglecting physical or mental symptoms and needs as they were busy balancing work and family responsibilities:

*“I would say that I don’t think I worry enough. And I’m not sure if it’s because, number one, being a woman, a working woman who will ignore physical pain or tiredness and just choke it up to well, maybe I had a long day at work, or a stressful week.” (119)*

#### Shame.

Young women detailed how the AMI diagnosis carried stigma. As having an AMI was perceived by the public as only targeting the elderly and those leading unhealthy lifestyles, women expressed feelings of **shame** and worthlessness. One woman tended to deny or avoid talking about her AMI for fear of being stigmatized:

*“I won’t go to a grocery store that’s near my place because I don’t want people who know me from the past to see me. I have to tell myself to pick my head up when walking and stop looking at the ground. I want to be invisible and I just don’t want people to see me… I limited who else I told because I figured if I told people they would say, ‘Well, she’s fat and overweight she deserved to have a heart attack.’ I never told anyone that because I felt they would just make fun of me. I felt like they’re looking at me as this overweight, middle aged white woman who didn’t matter, I guess I wasn’t worth it” (14)*

### Loss of hope and optimism

The feelings of guilt and shame for being weak, accompanied with difficulty in sustaining health behaviors, sometimes led to young women to feel hopeless.

*“After my second heart attack, the reason why I really needed therapy was because I wasn’t suicidal, but I wanted to get away from me. I wanted to leave what was going on.” (121)*

Their feelings of lack of hope and optimism were also magnified by the COVID-19 pandemic. One woman detailed how COVID-19 contributed to her loss of motivation in lifestyle changes.

*“Plus, I slowed down my life a lot, so I’m home a lot more now. So, I think like I just come home and I’m like, okay, when can I go to bed? The weight gain doesn’t help either. And then I also think too that the COVID thing definitely attributed to it as well. Because I think a lot of us lost our motivation during that period of time. It’s just hard to pick it back up now.” (114)*

### Loss of social roles

Young women described losing established roles in their families, communities, and occupations as a result of AMI. One woman explained how she could no longer take care of her family as a consequence of AMI. Her role as a caregiver was lost and her identity taken away:

*“I’ve always been like the stronger of my siblings. So now they realize that something could happen to me. I’m not as strong as everybody thinks. So they don’t depend on me as much as they used to because that was also causing a lot of pressure and stress. They’ve been taking care of themselves, especially my brother. He’s been holding his own now.” (63)*

#### Feeling infantilized.

After the AMI, young women also mourned the loss of independence and often struggled with feelings of dependency on others, summarized as **feeling infantilized**:

*“My family was really very supportive [but] very nervous, though. They didn’t want me to do anything. They did not want me to go back to work and they actually questioned my position, you know, making sure that it was okay to go back there. My husband didn’t want me to lift a finger or do anything and then my mother also came over and checked on me every day because my husband had to be at work.” (119)*

#### Feeling abandoned.

Young women often **felt abandoned** after experiencing an AMI, despite having family and friends with supportive intentions. One participant described how although she was able to be cared by her husband, she felt lonely because her husband could not relate her sentiments from a woman’s perspective:

*“I was very much alone which is scary. My husband had to go to work. He stayed at home the first two days. And it was winter, which was worse because I couldn’t walk outside. I was just lonely maybe and wanted someone to talk to about all this stuff that just happened. Just someone to, ‘Hey how are you doing today?’… I can talk to my husband, [but] he’s a guy and doesn’t really get it. He’s like, ‘You’re good, you’ll be tough. You’ll do it.’ You know, that’s not what I need to hear. I want a little bit of sensitivity, another woman’s perspective maybe” (160)*

#### Feeling invisible.

In addition, women **felt invisible**, and that their struggles remained unvalidated. As young women appeared physically “better” and “healthier,” others may have the misconception that “you’re ok, you’re alive, you’re fine.” However, this was often not the case. The feelings of being misunderstood and the resulting lack of compassion were expressed by participants:

*“I mean, everybody hears about the men having the heart attacks, the chest pain, but women don’t talk about anything. It’s like we’re supposed to be superwoman and supermom. There’s just so many things out there that I’m sure I still don’t even know about.” (157)*

#### Treated worse than men.

As a result of living in a gendered society, women also felt being **treated worse than men**. One woman described receiving differential responses in a working environment compared to a male coworker who also had an AMI:

*“One of the other teachers at work had a heart attack, like two weeks before I did, but he was more fit and everyone really rallied around him. [But when] I got back to work a couple weeks after having a heart attack, they wanted me to go up to Mohegan Sun with a unified sports group of students and I’m like, ‘I’m just trying to get through the day,’ and they were like, ‘Well, you’re the unified coach of the Year, you should get up there,’ and I’m like, ‘That is really insensitive.’ Whether it was a male-female thing I don’t know.” (14)*

#### Not being believed.

Young women also cited negative or even traumatic experience during their hospital care. One women was **being dismissed** in a clinical setting:

*“They put me out. They didn’t have room so they left me in a hallway. I thought I was going to die there.” (14)*

Several women had the experience of **being falsely accused**, when doctors were not able to identify a typical AMI in them and assumed alternative explanations such as drug use:

*“Actually, while I was in the hospital, I ended up having a cardiac catheterization and the doctor basically told me that they didn’t find any blockages and he just said that, ‘Are you on taking any [substances]?’” (34)*

*“I had a real bad experience with the doctor. Because I have multiple tattoos she was like, “Well, how much cocaine did you do?” Huh. I’m a grandma. I don’t do drugs. What are you talking about? I didn’t appreciate at all.” (37)*

Young women described **not being believed** that they had an AMI, as their struggles remained unvalidated. One woman detailed how people around her showed indifference to her AMI:

*“I think people are actually a little dismissive of it because my diagnosis wasn’t so bad. And because I didn’t die. They had, you know, if it kind of can’t kill me. But they don’t understand.” (57)*

## Gains

Four main themes were identified under the category of gain – young women described that the AMI had enhanced connection, gave them strategies to manage emotions, helped find meaning and purpose of life, and instilled a sense of hope and optimism. Since the “connection” (strengthened relationships) and sense of “hope/optimism” themes are closely aligned with what have been elucidated in prior work [23], we described the following two new themes specifically observed among young women.

### Strategies to manage emotions

While young women experienced shifts in mood after the AMI, they also described using different strategies to manage/cope with their strong emotions:

*“I just made that my goal, I’m not going to stress about the things that I can’t control at work. I’m not going to stress about, this boss being upset about something that I had no control over. I’m not going to let you take me there where I’m going to end up having another heart attack.” (63)*

One women also expressed how she used to be an emotional person, but a reframing of perspectives after the AMI helped her better manage emotions.

*“I try to stay in a very relaxed setting at all times, I don’t let myself get too worked up or too agitated with people. I’ll walk away from a situation whereas before, you know, I’m Italian and like we stand in our ground. And I just, I’m a Leo. So, like I said, I don’t let myself be in those type of predicaments.” (82)*

### Meaning and purpose

#### Prioritizing oneself.

Women also described how they often put others first than themselves, as the nature of their occupation or just being a woman. But after AMI they learned to take care of themselves first:

*“Because I used to work in the human services, I worked in group homes. So, you know, my whole life is about taking care of everybody else. I think that’s the issue with a lot of women. You know, we’re so busy taking care of everyone else, but we don’t take care of ourselves.” (114)*

One woman reflected on her past behavior and current commitment to prioritize her own health:

*“I wasn’t great about going to the doctor. I have to be now…You know, we’re so busy taking care of everyone else, but we don’t take care of ourselves. Plus a lot of poor life choices, you know, smoking, not eating, eating horribly, because I was always on the run all the time. I’m not on the run all the time now. It allows me to take better care of myself. (114)”*

## Discussion

Using a participatory, phenomenological approach and following the personal recovery framework, the present study contributes to the knowledge gap in the lived experience of young women recovering from an AMI. Findings validated the utility of personal recovery framework in capturing the experience of young women with AMI. Further, themes identified from the narratives of young women, which anchors around social roles and personal identity provide novel insights into their recovery experience. These findings are important in helping clinicians understand young women’s needs, tailoring secondary prevention strategies to improve outcomes among this high-risk group, and may inform the development of a personal recovery orientation in cardiology.

Prior qualitative studies of women of all ages focusing on post-AMI recovery have demonstrated that women have an overwhelming sense of uncertainty during their recovery [32–35]. Other studies regarding young women (18–55 years) suggested that they did not accurately assess their risk, reported poor preventive health behaviors, and delayed seeking care for symptoms during the acute phase [19]. Our study is the first attempt to understand the long-term recovery experience of young women with AMI under a patient-centered recovery lens. Young women have emerged as a group in need of special attention as they have worse outcomes compared with similar-aged men despite less severe disease; [11,15] and thus our study fills in an important research gap.

While the established personal recovery framework speaks to the experience of general AMI survivors, it was previously unclear whether this framework can be applied to young women with AMI. Evidenced from our research, the overall structure of young women’s experience did not deviate largely from the personal recovery framework, yet certain aspects were highlighted and further articulated in the current study, with an emphasis on young women’s psychosocial well-being and needs. First, young women described feeling self-blame and shame as cardiac events are less common and less understood in this group. Stigma or perceived judgment associated with AMI diagnosis may be particularly critical for young women feeling heard in the clinical space and supported in the recovery process. Due to physical and medical limitations after AMI, they became dependent on others to aid them in their everyday life, which created a feeling of being a burden on others, an emotional response recognized by prior research on middle aged and older women with AMI [36,37]. This sense of loss in self-worth has also been described in qualitative studies of younger adults aged <55 years [38] and younger men aged <49 years [39] with AMI. Young women in our study often returned to their job and housework early in their recovery, yet they expressed feelings of being debilitated and exhausted, which may suggest a lack of social support for this vulnerable population in order to sustain a modified lifestyle.

Second, during their recovery, young women’s own needs were not a priority because of a variety of competing demands and circumstances. Often, young women are an essential part of the family structure and struggle in recovering to the full capacity of working and taking care for others [40]. Young women in our study expressed concerns in fulfilling family and work responsibilities after AMI and some were forced to get back to work despite against medical recommendations. These competing and sometimes conflicting priorities have been previously reported among young women when appraising their symptoms and making care-seeking decisions before and during an AMI [19]. The mindset of “taking care of others instead of myself” has been persistent in younger women’s experience throughout the continuum of care, and indicates a critical need to develop targeted educational campaigns for these women to prioritize their own health in terms of managing traditional risk factors and psychosocial well-being [40].

Third, young women with AMI faces particular challenges with the healthcare system through their recovery. Women tend to describe unpleasant, if not traumatic experiences with the healthcare system. They received inconclusive results for AMI diagnosis tests, worse treatment compared to similar-aged men, and less clear answer as to what causes their AMI. They also discussed absence of cardiac rehabilitation (CR) facilities that fits their specific needs, in terms of the educational content, schedules, and peers. In our conversation with young women, it is commonly proposed to implement support groups for sharing the voices of young women and building a community. It is likely that caregivers and providers did not receive adequate information regarding the care and support required by these women, which warrant further research and implementation efforts to identify and improve.

Clinical and public health implications for these findings were substantial. Awareness of the new themes identified, particularly the psychosocial impact of AMI on young women, could highlight areas for clinical intervention. For example, improved health literacy around risk factors, prevention strategies, and identification of ‘atypical’ cardiac emergency symptoms may improve outcomes of young women post- AMI. Education in the broader community and the workplace about the recovery process following an AMI is also imperative. Next, issues highlighted in this study as particularly important to young women’s AMI recovery needs to be integrated into clinical practice. Clinicians may consider offering guidance to younger women on topics such as physical activity, housework, and emotion well-being. Similarly, cardiac rehabilitation (CR) facilities could be adapted to provide better support for younger women by offering evening/home-based care, considering the fact that these women often face more daily life pressure and juggle multiple professional and personal responsibilities. Lastly, it is imperative to provide comprehensive support, encompassing access to psychological assessment and tailored interventions that is not burdensome to the young women (e.g., WOOP [41,42]), counselling, smooth return-to-work programs, and peer support networks. It is important for the healthcare system to provide a holistic view with focus on psychosocial-related aspects to help accelerate young women’s recovery after AMI.

### Limitations

First, this study sampled younger women from diverse socio-economic backgrounds in three urban and suburban settings within a high-income country and focused on the experience of younger women aged <55 years old. The specificity of this setting should be kept in mind when considering results in a wider context. Second, as a result of conducting virtual interviews via Zoom due to the COVID-19 pandemic, participants may be more likely to provide socially desirable responses or may be less comfortable sharing sensitive information via Zoom. Further, the unique circumstances of the pandemic, including changes in social, economic, and healthcare systems, may affect participants’ recovery experience, and may limit the generalizability of the study findings to other periods of time.

## Conclusions

Using a participatory, phenomenological approach and following the personal recovery framework, the present study contributes to the knowledge gap in the lived experience of young women recovering from AMI. Findings validated the utility of personal recovery framework in capturing the experience of young women with AMI and highlighted themes that address young women’s distinguished ways of meaning-making, which anchors around social roles and personal identity. These findings are important in helping clinicians understand young women’s needs and preferences, tailoring secondary prevention strategies to improve outcomes among this high-risk group, and may inform the development of a personal recovery orientation in cardiology.

## Supporting information

S1 Text Patient enrollment flow chart and criteria. (DOCX)

S2 Text Patient interview guide. (DOCX)

S3 Text Quotes according to themes and subthemes. (DOCX)
