# Supplementary material for: Young women’s experience of personal recovery following acute myocardial infarction: A qualitative study
Source: PLoS One. 2025 Sep 9;20(9):e0298798. doi: 10.1371/journal.pone.0298798 (PMC12419669; doi:10.1371/journal.pone.0298798)
Supplement: S1 Text — (DOCX) [file pone.0298798.s001.docx]

**Supplement Material 1. Patient enrollment flow chart and criteria**

Young women with AMI identified

N=265

**Not eligible**

Physician adjudication without AMI: 116

No contact information: 113

Non-English speaking:1

Eligible young women

N=35

**Not interviewed**

Declined to participate/Lost to follow-up: 5

*An additional 12 young women were not interviewed as saturation was achieved.

Young women interviewed

N=18

Acute myocardial ischemia diagnosis was defined as either a Type 1 (atherosclerosis/thrombosis) or Type 2 (oxygen supply/demand imbalance) AMI based on criteria stated in the Fourth Universal Definition of Myocardial Infarction.(39) These participants must have had elevation in cardiac troponin to >99th percentile of the upper reference limit, **and** at least one of the following signs and/or symptoms of clinical myocardial ischemia: (1) symptoms of acute myocardial ischemia (such as chest pain, dyspnea or fatigue); (2) electrocardiogram changes indicative of new ischemia (such as new ST-T changes, new or presumably new left bundle branch block, or the development of pathological Q waves), or (3) other evidence of myocardial ischemia by imaging (such as new regional wall motion abnormalities on echocardiography). Patients were excluded if they were (1) unable to read or understand English, and (2) unable to provide informed consent.
