# Supplementary material for: Young women’s experience of personal recovery following acute myocardial infarction: A qualitative study
Source: PLoS One. 2025 Sep 9;20(9):e0298798. doi: 10.1371/journal.pone.0298798 (PMC12419669; doi:10.1371/journal.pone.0298798)
Supplement: S2 Text — (DOCX) [file pone.0298798.s002.docx]

**Patient Interview Guide**

Reminder to Interviewee: check that all potential distractions are eliminated such as: cell phone and computer calendar reminders are turned off, cell phone is in silent or do not disturb mode, make sure device for recording interview is plugged in and has a stable internet connection, remind participant they provided consent to participate in this interview, as a reminder it will be recorded for analysis of the conversation, the video is optional but to obtain as close to an in-person interview it would likely be best conducted with video, the video is not being used or analyzed, confirm you are recording before starting.

Introduction:

Good Morning (Afternoon, Evening), I want to start by thanking you for your time. The information you will share with me today will be very important in helping our research team. We have been talking to women to understand what life is like after having a heart attack. Our goal is to develop programs to help support women after they’ve had a heart attack to remain healthy and out of the hospital.

1. How have things been going for you lately?
2. What was having a heart attack like?
   1. What was life like after you had a heart attack?
   2. How did other people in your life react to your heart attack?
   3. Did you find yourself wondering why you had the heart attack? What was that like?
3. What’s it been like to continue to have trouble with your heart?
4. How have things been with your doctor?
   1. How has your relationship with your doctor been?
   2. Do you feel comfortable asking questions and discussing issues important to you with your doctor?
5. What’s been most important to you since the heart attack?
   1. What’s helped you do these things?
   2. What’s gotten in the way?
6. How has your daily life, day to day activities, been since the heart attack?
   1. Are there things that get in the way?
   2. Are they any tasks you no longer feel comfortable doing or able to do?
   3. What helps in your day to day activities?
   4. Did you make any lifestyle/behavior changes? Of some of these things that you changed, which were the hardest for you to keep up with?
7. Often people are told to change parts of their lives, like quitting smoking, doing physical activity, and taking prescriptions after a heart attack. Some women say they have trouble with these changes and some women express other problems like anxiety, depression, lack of emotional support, not working again, or access to care.

. Do any of these changes or problems fit your experience?

- 1. What are we missing? [prompt: are there other issues that we did not mention that were important in your recovery?]
  2. What was it like to have set all these expectations for yourself?
     1. Are your own expectations for yourself stressful? What’s that like?

1. Is there anything else you’d like to tell us about your experience in returning to hospital after your heart attack? Are there any things that would have been helpful to you?
2. If you had one wish for yourself, what would it be?

**Questions on checklist that may be answered above (prompt if not already discussed):**

1. Did any of the following factors contribute to you being readmitted and/or returning to hospital after the heart attack?
   1. Anxiety
   2. Depression
   3. Poor general health / more physical limitations / poor quality of life
   4. Lack of emotional support and social activities
   5. Obesity
   6. Smoking
   7. Social determinants of health (i.e. socio-economic status)
   8. Non-working status

2. How has your work status changed since the heart attack?

a. [If identified as working prior to the heart attack and still out]

What does working outside the home, having a job, mean to you?

1. How could we support you in getting back to work outside the home?
2. Did not working outside the home, having a job, contributed to going back to hospital?

b. [If ‘working’ at home (e.g. caregiving, housework)] How has your role at home been impacted since the heart attack?

3. Which provider do you consider as your primary doctor that takes care of you for medical events, when you get sick, not feel well?

4. Do you feel that the attitude of your healthcare providers changed after your heart attack?

5. Did you feel supported by providers?

6. Did you feel supported by your family, friends and community after the heart attack?

7. COVID specific question?
