# Supplementary material for: Young women’s experience of personal recovery following acute myocardial infarction: A qualitative study
Source: PLoS One. 2025 Sep 9;20(9):e0298798. doi: 10.1371/journal.pone.0298798 (PMC12419669; doi:10.1371/journal.pone.0298798)
Supplement: S3 Text — (DOCX) [file pone.0298798.s003.docx]

| **Framework** | **Themes** | **Sub-themes** | **Quotations** |
| --- | --- | --- | --- |
| **Shift in identity -**  **‘I’m a different person now’** | Active process |  | *“[Life after AMI is] Very crazy because I tried to do things so that way, I won't have another heart attack. But it's kind of difficult. When you're working and you're doing other things, you never get that time to yourself to really relax and really rest yourself. So it's like you trying to keep eye out. Eat healthy. Take your medications.” [Y70]*  *“I've learned that if I want to plan out my week I realized that that's too broad of a range. I have to take it day by day [and] how I'm feeling at the start of the day will dictate what it is that I can accomplish for that day. I do my very best to, you know set a schedule, making sure that I'm eating a light breakfast, staying hydrated during the day, making sure that I'm taking my medications. Getting in, even if it's the slightest physical activity, like walking, because although I'm a nurse, I do a lot of administrative work now.” [Y119]* |
|  | Accepting limitations |  | *“I get tired a little bit easier, I call it feeling my bones. I'll push myself, but I also know when my heart is telling me it's enough. I usually wear a smartwatch and if I'm jogging and my heartbeat goes over 145, then I know I need to back it off a little bit. [In terms of daily tasks,] I'm doing more now than I used to. Just I'm more careful with it.” [Y157]*  *“I worked at XX Hospital and I worked as a travel nurse, but I had to limit my travel. So, those things that actually happened after I had a heart attack I kind of limit how far I would go away from home. I mean, I've been working 48 [hours] and I just noticed like, I'm like super tired at work. So I'm like, I have to cut back. I have to go down to 36. It was like a huge warning like it was that big yellow sign at the end of the road like “Don't go any further.” The working part. I was very concerned.” [Y34]* |
|  | Responsibility |  | *“Everything else can be at hold but when it comes to the children and the food and a roof over your head that's what you think about, you know, just to make sure that you keep that. I've been lucky enough to keep a job during this epidemic. No, I actually stopped the driving because that was very stressful. It was at least 14 to 16 hours a day that I was doing. Yeah, so I got myself a local job. I'm working in a factory and a bakery and I do quality control there.” [Y170]*  *“Now I don’t eating if it's not healthy because I'm about to have a grandchild number 14 in June. I want to be able to see them. I am gonna hang around as much as I can.” [Y37]* |
| **LOSS** | Safety and security | Fear | *“[Life after having a heart attack is] Scary, because I didn't have any answers. Very scary… [So basically, you came back to hospital, you know, because you're too scared about what was going on and you needed to some answers then.] ” [Y34]*  *“So it's like you trying to keep eye out. Eat healthy. Take your medications. You worry about every little thing. I can't run. Like not run, run, but jog. Every time you feel a pinch, it could be anything. But the pinch make you worry that you need to go sit down. I now have a new way of doing things, you know, to modify.” [Y70]*  *“I feel like I'm going to pass out full. And that’s it. And it's very scary. You know, once you have a heart attack, you're afraid that it's going to happen again. I'd give birth every year all natural. And my kids are 10 pound babies.” [Y82]* |
|  |  | Feeling overwhelmed | *“I dealt with and am dealing with depression. It was hard because [my psychiatrist gave me] more medication. Why do I have to be so young taking all this medication? I had all of these situations going on in my life that would cause any normal human being to become depressed. I need to be able to function like a normal human being without feeling so angry all the time. I want to scream or yell or cry. [I was] just not happy and not in a good space. It was hard for me to come to terms with the fact that this was something else that I was going to need to be treated for. It’s just really overwhelming with all that I have going on, and trying to maintain my heart health and not get stressed and overwhelmed with it.” [Y105]*  *“Whereas the first surgery it was hard, but it was nothing like this. Right now, I just can’t seem to get a hang on the things. It’s like they got me taking Xanax and at this point, I know that I need the Xanax. You know, it’s like I can’t control my feelings and emotions. I just feel like a totally different person.” [Y16]* |
|  |  | Financial insecurity | *“Yeah, I have to go back to work. There was no question or even if the doctor told me that I couldn't go back to work there was no -- the bills can't get paid without me working. It is difficult for somebody like me that doesn't have a support system to be able to come out from the hospital and then, you know, have this activities that you're supposed to have, but then cannot because of your financial situation or whatever situation you might have. Everything else can be at hold but when it comes to the children and the food and a roof over your head that's what you think about, you know, just to make sure that you keep that.” [Y170]*  *“They both times recommended cardiac rehab. The first time I went three visits and could not afford [it]. That adds up to three visits a week, it's a lot of money coming out of our paychecks. I still have the same insurance and [and] I actually turned one down [an echo] because I knew it was going to cost me another $1,000. I'm like, no, I can't. I talked to [the new cardiologist] like, listen, if I buy this Fitbit and I send you my information once a month, can we call that cardiac rehab? And he agreed to it.” [Y157]* |
|  |  | Difficult to sustain behavioral changes without support | *“I did [try and quit after the heart attack], and I did quit for like about three months, you know, after I came out of the hospital, but then I started hanging out with the people that still smoke and, that came back around and, it's hard. It's really, really hard and fortunately, I don't have anybody that smokes in the house, but then my coworkers and friends are smoking. You work with them or you, you know, so yeah, it doesn't help” [Y170]*  *“I can talk to my husband, [but] he's a guy and doesn't really get it. He's like, “You're good, you'll be tough. You'll do it.” You know, that's not what I need to hear. [I want] a little bit of sensitivity, another woman's perspective maybe.” [Y160]* |
|  | Self-worth | Self-blame | *“It has to do with my smoking. Just marijuana and cigarettes that will be it. It's bad enough with the two. If I quit - it will help a lot. [Quit smoking] It’s horrible. It's horrible especially that I've been smoking for so many years.” [Y170]*  *“When the MS kicked in, I kind of dropped the ball with the cardiac stuff. And I should have been more proactive with [managing my cholesterol]. Even though I was taking the Lipitor, my numbers were still high. So I should have been on more medication, which I am now.” [Y105]*  *“ I think it was my own doing to have to come back because I still was in denial. I still wouldn't say that I had a heart attack.” [Y70]*  *“Prior to having my heart attack, the doctors were giving me medicine [for my] cholesterol. [They said] “you need to take this.” That didn't help that they're giving me the medicine and I'm not taking it. So I kind of like basically did myself in because I wasn't doing what I was supposed to do.” [Y37]*  *“I definitely know what brought me there. I was stressed out. They say it and you've heard it a million times stress will kill you. I mean, I have to say I agree with that, I have to say.” [Y57]*  *I felt like they're looking at me as this overweight, middle aged white woman [who] didn't matter, I guess and I wasn't worth it.” [Y14]* |
|  |  | Self-neglect | *“I don't think I worry, or that it causes anxiety. I would say that I don't think I worry enough. And I'm not sure if it's because, number one, being a woman, a working woman who will ignore physical pain or tiredness and just choke it up to well, maybe I had a long day at work, or a stressful week.” [Y119]*  *“So, it's just been a really hard year. I don't exercise. I really just work all day. Between virtual lessons and this and that it just takes up all of my time and I just have not had time to take care of myself.” [Y14]* |
|  |  | Shame | *“I still wouldn't say that I had a heart attack. I would say, “That's what they said.” I think because I didn't want to say that my health is failing. I didn't want to feel like you know, heart attacks happen to old people. So I’m getting old. And I just didn't want nobody to treat me like I was feeble or like, I can't do.” [Y70]*  *“[Right now] I won't go places. I won't go to a grocery store that's near my place because I don't want people who know me from the past because I don't want them to see me. I have to tell myself to pick my head up when walking [and] stop looking at the ground I want to be invisible and I just don't want people to see me… I limited who else I told because I figured if I told people they would say, “Well, she's fat and overweight she deserved to have a heart attack.” I never told anyone that because I felt they would just make fun of me.” [Y14]*  *“I've got a few friends that like every once in a while, they'll checkup or the close ones that we hang out, we joke about [that] day. But most of them seem to not want to talk about it. It's like taboo subject. [Sometimes they’re like] “hey, you're too young to have that” or the blame game “well, it was because you were a smoker.” [Y157]* |
|  | Lack of hope and optimism |  | *“After my second heart attack, the reason why I really needed therapy was because I wasn't suicidal, but I wanted to get away from me. I wanted to leave what was going on.” [Y121]*  *“I think between the quitting smoking, the beta blocker, and the menopausal thing, the weight is like not good. It’s very discouraging. And, I did that before I had the heart attack. See? I quit smoking, I tried to eat healthy, and I had a heart attack. Plus, I slowed down my life a lot, so I'm home a lot more now. So, I think like I just come home and I'm like, okay, when can I go to bed? The weight gain doesn’t help either. And then I also think too that the COVID thing definitely attributed to it as well. Because I think a lot of us lost our motivation during that period of time. It’s just hard to pick it back up now.” [Y114]*  *“I've never been a depressed person at all. I'd say that you're always fighting a depression after this. If I don't exercise every single day, I feel like that throws me into a depression. It’s almost like I need my routines to know that I'm doing the right thing. Otherwise, I'm disappointing myself, disappointing my family. I had never had a panic attack in my, but I put myself in the emergency room twice thinking I was having another heart attack.” [Y157]*  *“I’m walking in pins and needles and got to think of the worst and hope for the best.” [Y68]* |
|  | Loss of social roles | Feeling infantilized | *“If I go to lift something, there's always somebody at work going “you're not supposed to do that. Put that down.” [Y63]*  *“My family was really very supportive [but] very nervous, though. They didn't want me to do anything [during my recovery]. They did not [want me to go back to work] and they actually questioned my position, you know, making sure that it was okay to go back there. My husband didn't want me to lift a finger or do anything and then my mother also came over and checked on me every day because my husband had to be at work.” [Y119]* |
|  |  | Feeling abandoned | *“[Sometimes I felt like] I was very much alone which is scary. My husband had to go to work. He stayed at home the first two days. And it was winter, which was worse because I couldn't walk outside. [I] was just lonely maybe [and wanted] someone to talk to about all this stuff that just happened. Just someone to, “Hey how are you doing today?” [Y160]*  *“having no one to talk to, other than Google and you know. And you know, all of your stages of recovery, there's really not really somewhere that I could figure out somewhere to go” [Y57]* |
|  |  | Feeling invisible | *“Other people, like, say, for instance, my coworkers, almost didn't believe that I had [a heart attack]. The first thing that you know, people say is, “Oh, you're too young. Are you sure that's what you had?”. [Now] I stress that it doesn't care how old you are really. It's just something that people really don't hear a lot about it happening, so they don't really believe that it happens.” [Y119]* |
|  |  | Treated worse than men | *“I mean, everybody hears about the men having the heart attacks, the chest pain, but women don't talk about anything. It's like we're supposed to be superwoman and supermom There's just so many things out there that I'm sure I still don't even know about.” [Y157]*  *“One of the other teachers at work had a heart attack, like two weeks before I did, but he was more fit and everyone really rallied around him. [But when] I got back to work a couple weeks after having a heart attack, they wanted me to go up to Mohegan Sun with a unified sports group of students and I'm like, “I'm just trying to get through the day,” and they were like, “Well, you're the unified coach of the Year, you should get up there,” and I'm like, “That is really insensitive.” Whether it was a male-female thing I don't know.” [Y14]*  *“I had a real bad experience with the doctor. Because I have multiple tattoos she was like, “Well, how much cocaine did you do?” Huh. I'm a grandma. I don't do [take any illegal substances] drugs. What are you talking about? I didn't appreciate at all.” [Y37]*  *“And then I went to the ER. And they told me that I had bronchitis and I'm looking for something for pain and he's telling me, ‘You don't need anything for pain. I could give you Tylenol, but you're not getting anything else.’ And I was just like, ‘Jesus Christ, you're treating me like as if I'm a drug addict or something.’ It's very upsetting. Yeah, we live in Fair Haven. And, you know, I know there's a lot of case scenarios that are different in Fair Haven. But not everybody that lives in Fair Haven is a drug addict.” [Y82]*  *“I mean, everybody hears about the men having the heart attacks, the chest pain, but women don't talk about anything. It's like we're supposed to be superwoman and supermom There's just so many things out there that I'm sure I still don't even know about.” [Y157]* |
|  |  | Not being believed | *“So supposedly, that's is one of the worst heart attack. You know, and because I was in such a stressful environment. I was actually at work when it happened and I just had to literally like stop and brace myself on the side of my desk, for whatever it was that you know, was hurting me. I didn't even know that's what was happening at first, I just felt pain. And my boss telling me just push through, it's probably just anxiety or stress or something. I said “I don't suffer from anxiety.” [Y82]*  *“I think people are actually a little dismissive of it because my diagnosis wasn't so bad. And because I didn't die. They had, you know, if it kind of can't kill me. But they don't understand.” [Y57]* |
| **GAIN** | **Connection** | Strengthened relationships | *“It's hard on the marriage. And I've been married three years now to a wonderful man, who's been through both of them with me. But he's five years older and is an 18-year-old at heart. So to try and keep up the way I used to is harder now. [We got married] shortly after the second one. That one [made him] more aware of me medically. Like before he wanted to hear nothing about my doctor's appointments. Now, he's really more involved in it. That makes me feel good. He's a rare one. He could have just dropped me and ran hard, but he didn’t.” [Y157]*  *“I did have a guy that I was living with during this time and before I went in the hospital, everything was all fine. And I recently -- you know, just getting a new heart trying to heal and stuff like that. You don’t want to go through relationship issues with a broken heart and stuff like that. So now I’m at a point where I have to protect my own heart. I have to do it myself. [AMI] has affected a lot of my relationships as well. So it’s affected me in a way where I’m so clear, right now, that I see everything and everyone for who they are.” [Y16]*  *“Yeah, well, it's funny, I actually ended a few 35-year friendships after the heart attack. But it puts a total reset on your life. And you have to reevaluate what's important.” [Y57]* |
|  |  | Return to prior activities | *“I did continue to work [after AMI]. That's just who I am. It's just in my nature. I was working up until I got sick with COVID last year. Unfortunately, right now, with everything going on and I haven't gotten the vaccine yet. I'm scared to go back. I got really sick with COVID last year and I couldn't get off my couch for almost three weeks straight.” [Y82]*  *“It's the same routine work, home, cook, clean, take care of kids and back to the same thing the next day. Well, I do all that. Well, I've been doing it for so long. It's just a normal thing for me.” [Y170]* |
|  | Strategies to manage emotions |  | *“I just made that my goal, I'm not going to stress about the things that I can't control at work. I'm not going to stress about, this boss being upset about something that I had no control over. I'm not going to let you take me there where I'm going to end up having another heart attack.” [Y63]*  *“I try to stay in a very relaxed setting at all times, I don't let myself get too worked up or too agitated with people. I'll walk away from a situation whereas before, you know, I'm Italian and like we stand in our ground. And I just, I'm a Leo. So, like I said, I don't let myself be in those type of predicaments.” [Y82]* |
|  | **Meaning/Purpose** | Re-thinking values | *“I open my own business, because I'm sick and tired of pressure from outside. Because I have hydrocephalus, but it wasn't diagnosed until I was 15. So like I already had some of this like willy-nilly, fly by the seat of your pants, sort of attitude. And then heart attack was like I need to have fun and be present and enjoy. So yeah, I guess you could say I found me. I found a different me. I want to control the stress.”* [Y121]  *“[At work] I make sure that I get up and go walk at least every hour, you know, around the hospital or at the unit or stretch and things like that. Being healthy and staying active [has been] the most important thing to me.”* [Y119] |
|  |  | More present | *“It brought me right to the present moment in reality, that's for sure. Take care of yourself. I think that's what we learn in life, right? Is that no one else is going to do it. So it's uh, it puts that on, it puts that on us.”* [Y57] |
|  |  | Feeling grateful to be alive | *“I’m just realizing that I've been given the opportunity. I'm still here, I'm still living. So that that makes me feel like I have something to look forward to.”* [Y119]  *“I thought about life or how it could have been a lot worse. I could have died. So I kind of pace myself now and just try to relax, smell the roses, so to speak. I take that time now.”* [Y63] |
|  |  | Prioritizing myself | *“I've always been like the stronger of my siblings. So now they realize that, something could happen to me. I'm not as strong as everybody thinks. So they don't depend on me as much as they used to because that was also causing a lot of pressure [and] stress. They’ve been taking care of themselves, especially my brother. He’s been holding his own now.” [Y63]*  *“Because I used to work in the human services, I worked in group homes. So, you know, my whole life is about taking care of everybody else. I think that's the issue with a lot of women. We're so busy taking care of everyone else, but we don't take care of ourselves.” [Y114]* |
|  |  | Relief | *“I didn’t question why it happened to me, because I felt like it was going to happen eventually, because of all the heart problems that I was still having after surgery.” [Y16]*  *“it was expected because it came from both sides of my family, you know, so I mean, I don't know, I guess. [So, did you kind of feel then you were kind of waiting for it to happen and then it happened and then you're like, oh, whatever?] Exactly.” [Y170]*  *“I really didn't [wonder why I had heart attack] because honestly I've been diabetic since I was 17 and I was very bad in my 20s. I didn't take care of myself and I probably caused, or I could have prevented it if I had taken better care of myself younger. So, I just wish I had known that all this really is going to happen. It's just a matter of when. It's like, you know, you read about it, but you go, yeah, well, maybe it'll happen, maybe it won't. I am only 20. How could that be me? I wish I had taken it differently.” [Y55]* |
|  | **Hope/optimism** |  | *“I just turned 44 in January, and my first grandson was born January 6, so I want to be around to see this little guy grow up. So I want to be healthy and around for this little guy. Now that my children are all grown up, and having their own.” [Y105]*  *“I wish that this heart would keep me alive for the next 40 years. I want to be more active and I want a family again. I want little kids. My kids are 14 and 20. I’m still young, so I can have another baby or two if I want to. I’m ready for it. Whoever I wind up with married and children in the house and traveling, having fun, whatever.” [Y16]*  *“I would like to see the world. Yeah, I would like to go back to traveling. I'm back in California. Yeah, well, my plan was to always go somewhere warm for the summer -- I mean, for the winter, and then come back home in the summer. That used to be my plan for years.” [Y34]* |
